# The analgesic effects of quadratus lumborum block versus caudal block for pediatric patients undergoing abdominal surgery: a systematic review and meta-analysis

**DOI:** 10.3389/fped.2025.1492876

**Published:** 2025-02-06

**Authors:** Yu Zhu, Jin Wu, Shenglong Qu, Peng Jiang, Chetan Bohara, Yi Li

**Affiliations:** ^1^ Department of Anesthesiology, Affiliated Hospital of Jiangsu University, Zhenjiang, Jiangsu, China; ^2^ Department of Anesthesiology and Pain Management, Lumbini Medical College and Teaching Hospital, Tansen, Nepal

**Keywords:** quadratus lumborum block, caudal block, pediatric, rescue analgesia, postoperative pain scores

## Abstract

**Background:**

Since children cannot express pain, postoperative pain treatment for them is relatively lacking. In this meta-analysis, we compared the postoperative analgesic effects of quadratus lumborum block (QLB) and caudal block (CB) in surgeries involving the lower abdomen, inguinal region, and urogenital system in children.

**Objective:**

This review examined the postoperative analgesic effects of QLB and CB in pediatric patients (0–18 years of age) undergoing abdominal surgery. The primary endpoint was the rate of postoperative rescue analgesia, defined as the proportion of patients who returned to acetaminophen, ibuprofen, and other analgesics when the pain score was greater than the protocol preset value within 24 h after surgery. Secondary outcomes included resting pain scores (0–10) at 30 min, 4 h, 12 h, and 24 h after surgery. Other secondary outcome measures were the time of first rescue analgesia, the incidence of PONV, and the incidence of postoperative complications, such as post-block infection, anaphylaxis to local anesthesia and hematoma.

**Evidence review:**

We systematically reviewed Pubmed, Central, EMBASE, Google Scholar, Web of Science citation index, the US clinical trials register, and abstracts for randomized controlled trials that compared these blocks and reported the rate of postoperative rescue analgesia.

**Findings:**

Seven RCTs (444 patients) were included in the final analysis. In pediatric abdominal surgery, compared with CB, QLB could reduce the rate of postoperative rescue analgesia within 24 h after surgery (RR = 0.37; 95% CI = 0.26 to 0.51; *P* < 0.01). The pain score in the QLB group at 4 (SMD = −0.11; 95% CI = −0.21 to −0.01; *P* = 0.02) and 12 h (SMD = −0.11; 95% CI = −0.22 to 0.00; *P* = 0.06) after surgery was lower, but at 0.5(SMD = 0.42; 95% CI = 0.34 to 0.50; *P* < 0.01) and 24 h (SMD = 0.30; 95% CI = 0.03 to 0.58; *P* = 0.03) was higher than that in the CB group. Of note, these pain score differences were not clinically significant. In addition, there was no significant difference in the incidence of complications or side effects between the QLB and the CB group (RR = 0.94; 95% CI = 0.59 to 1.48; *P* = 0.77).

**Conclusion:**

In conclusion, QLB might have a better postoperative analgesic effect for lower abdominal surgery than CB in pediatric patients. However, due to the relatively few RCTs identified and significant heterogeneity, further research in the future is needed to prove these findings.

**Systematic Review Registration:**

identifier (CRD 42023441447).

## Introduction

In recent studies, there is limited evidence regarding postoperative analgesia in pediatric patients. Children are unable to express pain, resulting in a relative lack of postoperative pain treatment in children (1). Better postoperative analgesia can accelerate the recovery of children, alleviate their pain, shorten hospital stays, and improve parental satisfaction (2). Regional blockade is a crucial approach in the multimodal analgesia regimen for pediatric perioperative care, and caudal block (CB) has been extensively utilized for postoperative analgesia in pediatric patients (3). The widespread application of ultrasound-guided technology helps to accurately locate sacrococcygeal block, especially when children have sacral abnormalities and other issues (4). Meanwhile, some studies have shown that the use of CB can promote early postoperative mobility and hemodynamic stability in children (5). However, its application is gradually limited due to its short duration of analgesia and weakness such as urinary retention (6).

Currently, the quadratus lumborum block (QLB) has garnered increasing interest among both adults and children (7–10). There are four different types of QLB, including external QLB, posterior QLB, anterior QLB, and intramuscular QLB (11), which are in line with the relative position of the needle tip and the quadratus lumborum. The main mechanism of action of QLB (12–14) is that the lateral arcuate ligament not only serves as a connection between the thoracic fascia and transverse fascia but also provides a pathway for local anesthetics in QLB to spread to the thoracic paravertebral space, thereby alleviating somatic and visceral pain.

The present meta-analysis aimed to compare the postoperative analgesic effects of the QLB with those of traditional CB, including the postoperative rescue analgesic rate during 24 spread to the thoracic paravertebral space, thereby alleviatirates of postoperative complications, in order to identify the advantages and disadvantages of the two analgesic methods.

## Materials and methods

### Study objectives

The overall objective of this study is to compare the analgesic effects of QLB vs. CB in children undergoing abdominal surgery. The primary outcome is the postoperative rescue analgesia rate, defined as the proportion of patients requiring additional analgesics such as acetaminophen or ibuprofen when their pain score exceeds the predefined threshold within 24 h after surgery. Secondary outcomes include resting pain scores (on a 0–10 scale) at 30 min, 4 h, 12 h, and 24 h post-operatively, the time to first rescue analgesia, the incidence of postoperative nausea and vomiting (PONV), and postoperative complications (such as post-block infection, local anesthetic allergy, hematoma, nerve injury, and local anesthetic toxicity). This systematic review was registered under PROSPERO, ID CRD 42023441447.

### Search strategy

The primary literature search was performed in July 2023, and the PubMed, Cochrane Library, Web of Science, and Embase databases were searched from inception until July 2023. To ensure that no newly published article was dropped, the secondary literature search was conducted in August 2023. During the initial literature search, we searched the terms edQL OR quadratus lumborum OR QL block OR QLB OR quadratus lumborum block)” AND “(CB OR caudal block OR caudal epidural blocks OR caudal analgesia OR caudal blockade OR caudal anesthesia OR caudal regional anesthesia OR caudal extradural anesthesia)” AND “(pediatric OR children OR infant OR adolescent OR schoolchild OR preschool OR teens OR youth)”. The secondary literature search was carried out the same as the primary literature search to find new articles.

### Study selection criteria

This meta-analysis was conducted according to the Preferred Reporting Items for Systematic Reviews and Meta-Analyses (PRISMA) (6). The inclusion criteria were as follows: (1) Comparing the effects of analgesia between the QLB and CB for pediatric patients undergoing abdominal surgery; (2) Consideration of the rate of postoperative rescue analgesia as the outcome; (3) Randomized controlled trials (RCTs). The exclusion criteria were as follows: (1) Receiving additional treatment in the control or experimental groups; (2) Duplicate publication; (3) An indeterminate type of study or non-RCTs; (4) Failure to extract valid data; (5) Unavailability of the full text of study; (6) Conference abstracts; (7) Reviews or systematic reviews.

Two independent reviewers reviewed and identified studies based on the previously mentioned strategy. Any discrepancies were resolved by discussing them with a third reviewer.

### Data extraction

The data extraction was performed by two researchers. Some studies used graphs to show the outcomes in lieu of data, thus, it was attempted to contact corresponding authors to obtain original data. Nevertheless, if no reply was received from the corresponding authors for the original data, GetData tool was used to extract values for each data point.

The following data were extracted: first author's name, country, date of publication, study design, the sample size of experimental and control groups, participants' characteristics, surgical procedures, analgesic methods (medication type, dose, tube placement, duration), time of follow-up, the rescue analgesia, pain scores [time of evaluation, mean, and standard deviation (SD)], the incidence rates of postoperative complications or by-affects (e.g., PONV). For the quantitative analysis, the numeric rating scale (NRS) or the visual analogue scale (VAS) scores were calculated in the range of 0–10 points (0 = no pain, 10 = extreme pain). Pain scores reported as face, legs, activity, cry, consolability (FLACC), or Children's Hospital of Eastern Ontario pain scale (CHEOPS) were transformed to a scale ranging from 0 to 10 points.

### Statistical analysis

We conducted meta-analysis for all outcomes. The data was analyzed using Review Manager V5.3. (Cochrane Collaboration, Copenhagen). A weighted average difference or standard average difference was used to analyze the continuous data, and 95% confidence intervals (CIs) were used to combine different scales. Data were presented as mean, and SD values were extracted directly. Data were presented as median using the following formula: (a + m + b)/3, where a = Q25 and b = Q75 to convert median to mean, and data were presented as interquartile range (IQR) using the following formula: SD = IQR/1.349 to convert IQR to SD. Data were presented as CI using the following formula: 95% CI = x ± 1.96*SE, where SD = SE* √n to convert CI to SD. The above-mentioned statistical formulas were previously described by Hozo et al. (15). We used *I*^2^ statistics to quantify heterogeneity. If *I*^2^ ≤ 50%, we chose a fixed effects model; if *I*^2^ > 50%, we chose a random effects model.

## Results

### Description of included studies

**Figure 1:**
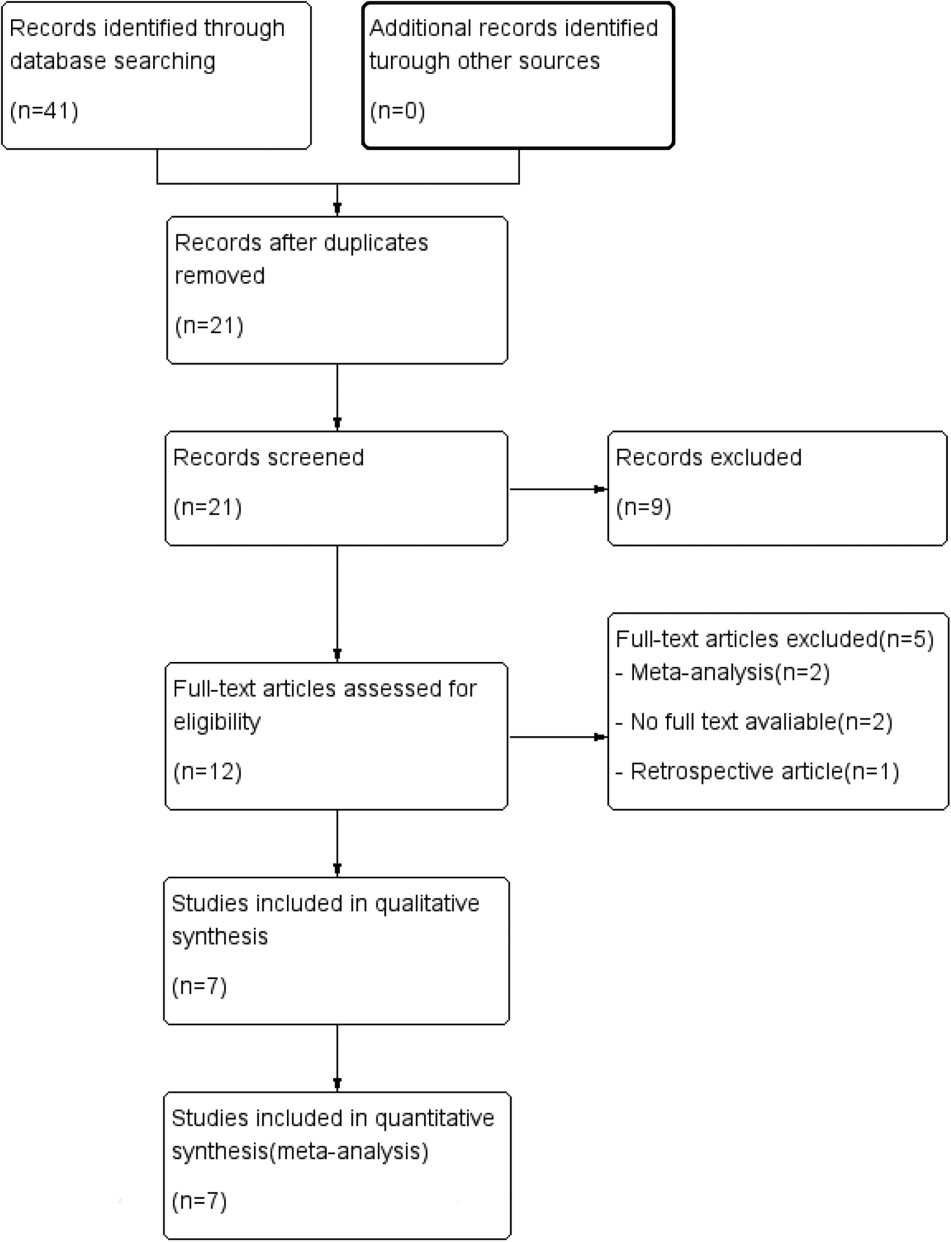
Search flow chart.

**Figure 2:**
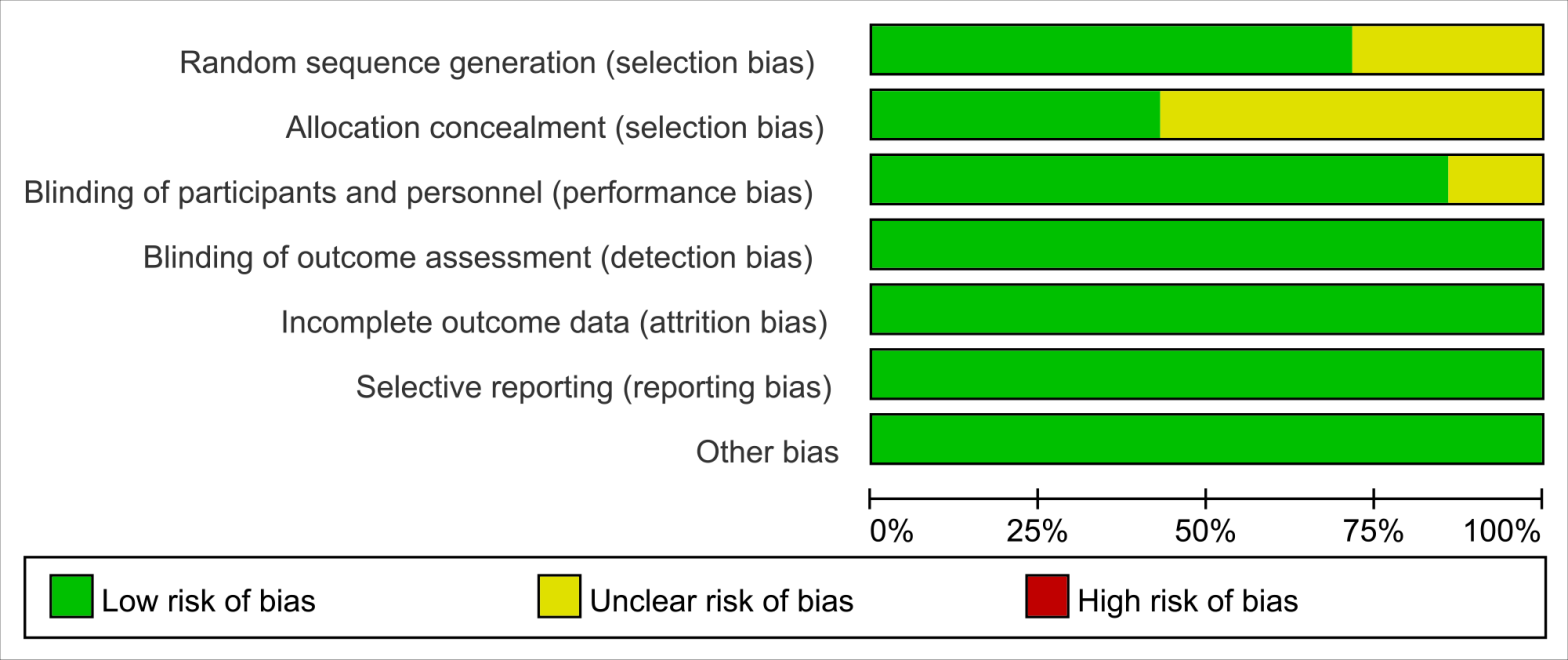
The risk of bias graph for included randomized controlled trials.

**Figure 3:**
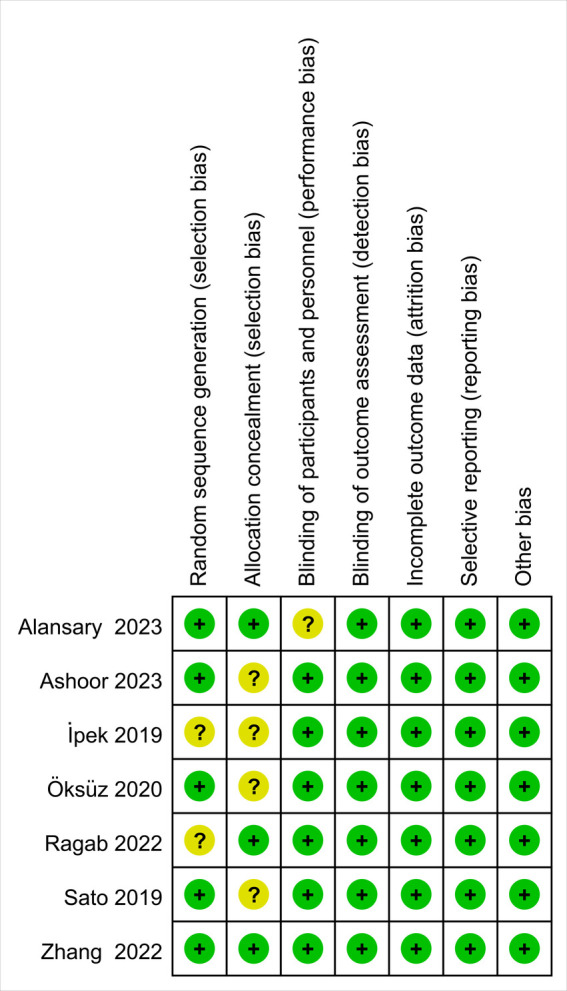
The risk of bias summary for included randomized controlled trials.

A total of 41 trials were identified in the primary literature search, and there were 21 of the records after duplicates were removed. Based on the inclusion and exclusion criteria, 9 trials were excluded, and 12 trials were further screened, of which 5 trials were removed (Figure 1) (13, 16–21). Characteristics of the included studies are listed in Table 1. A summary of findings is reported in Table 2. The risk of bias (RoB) assessment is reported in Figures 2, 3, with the randomization process and selection of reported results being the most common sources of potential bias.

**Table 1:** Characteristics of included studies.

Study	Patient age (year)	Surgeries included	Block	Number of patients(n)	Medicatios administered in block	Pain score at which rescue analgesia was administered	Rescue analgesia dose
Alansary et al. (16)	2–11	Open renal surgery	CB	20	1.25 ml/kg of 0.2% bupivacaine	FLACC ≥ 4	IV 0.5 mg/kg ketorolac
			QLB	20	1.25 ml/kg of 0.2% bupivacaine		
Ashoor et al. (17)	1–5	Inguinal hernia repair, Orchiopexy	CB	39	1 ml/kg of 0.25% bupivacaine + 2 µg/kg neostigmine	FLACC > 4	IV 15 mg/kg acetaminophen
			QLB	32	1 ml/kg of 0.25% bupivacaine		
İpek et al. (18)	0.5–14	Hydrocelectomy, Inguinal hernia, Orchiopexy, Orchiopexy + hydrocelectomy, Orchiopexy + İng. hernia	CB	30	0.5 ml/kg of 0.25% bupivacaine	In hospital: POAS > 5 At home: feel pain	In hospital:IV 10 mg/kg paracetamol; At home:PO 10 mg/kg ibuprofen syrup
			QLB	35	0.5 ml/kg of 0.25% bupivacaine		
Öksüz et al. (19)	1–9	Inguinal hernia repair, Orchiopexy	CB	25	0.7 ml/kg of 0.25% bupivacaine	FLACC > 4 or FLACC > 2	FLACC > 4 iv. 1 µg/kg fentanyl citrate; FLACC > 2 po. 7 mg/kg ibuprofen
			QLB	27	0.7 ml/kg of 0.25% bupivacaine		
Ragab et al. (20)	1–7	Hernia, Undescended testis, Hydrocele	CB	26	1 ml/kg of 0.25% bupivacaine	In hospital:FLACC > 4 At home:feel pain	In hospital:PR diclofenac sodium (1 mg/kg) At home:PO paracetamol (30 mg/kg)
			QLB	26	0.5 ml/kg of 0.25% bupivacaine		
Sato et al. (13)	1–17	Bilateral ureteral reimplantation surgery via a low transverse incision	CB	22	0.03 mg/kg morphine + 1.0 ml/kg of 0.2% ropivacaine	CHEOPS ≥ 7 or intermittent lower abdominal cramps associated with sensation of urgency to void	PNCA:bolus dose: 0.2 µg/kg, lock-out time: 15 min; Acetaminophen was administered intravenously 7.5 mg/kg to patients under 2 years of age and 15 mg/kg intravenously to patients 2–17 years of age in both groups every 6 h within 48 h
			QLB	22	0.5 ml/kg 0.2% ropivacaine		
Zhang et al. (21)	1–12	Genitourinary, General surgery	CB	60	1.0 ml/kg of 0.2% ropivacaine	FLACC > 4	IV tramadol 1 mg/kg
			QLB	60	1.0 ml/kg of 0.2% ropivacaine		

FLACC, face, legs, activity, cry, consolability scale; POAS, pediatric objective pain scale; CHEOPS, children Hospital of Eastern Ontario pain scale.

**Table 2:** Summary of findings.

Outcomes	Certainty assessment							№ of patients		Effect		Certainty	Importance
	№ of studies	Study design	Risk of bias	Inconsistency	Indirectness	Imprecision	Other considerations	QLB	CB	Relative (95% CI)	Absolute (95% CI)		
The rate of postoperative rescue analgesia	5	randomised trials	not serious	serious^a^	not serious	not serious	none	31/169 (18.3%)	90/169 (53.3%)	RR 0.38 (0.22 to 0.66)	330 fewer per 1,000 (from 415 fewer to 181 fewer)	⊕⊕⊕◯ Moderate^a^	CRITICAL
Pain score 30 min after surgery	4	randomised trials	not serious	very serious^b^	not serious	serious^c^	none	128	128	–	MD 0.24 higher (0.15 lower to 0.63 higher)	⊕◯◯◯ Very low^b^^,^^c^	IMPORTANT
Pain score 4 h after surgery	4	randomised trials	not serious	not serious	not serious	not serious	none	128	128	–	MD 0.11 lower (0.21 lower to 0.01 lower)	⊕⊕⊕⊕ High	IMPORTANT
Pain score 12 h after surgery	3	randomised trials	not serious	very serious^b^	not serious	serious^c^	none	106	106	–	MD 0.76 lower (2.03 lower to 0.51 higher)	⊕◯◯◯ Very low^b^^,^^c^	IMPORTANT
Pain score 24 h after surgery	4	randomised trials	not serious	very serious^b^	not serious	serious^c^	none	128	128	–	MD 0.12 higher (0.3 lower to 0.54 higher)	⊕◯◯◯ Very low^b^^,^^c^	IMPORTANT
Complications or side effects in the included studies	7	randomised trials	not serious	not serious	not serious	serious^c^	none	23/222 (10.4%)	27/222 (12.2%)	RR 0.94 (0.59 to 1.48)	7 fewer per 1,000 (from 50 fewer to 58 more)	⊕⊕⊕◯ Moderate^c^	IMPORTANT

CI, confidence interval; MD, mean difference; RR, risk ratioPopulation, pediatric surgical patients.

^a^ 50% ≤ *I*² < 75%.

^b^ *I*^2^ ≥ 75%.

^c^ The 95% confidence interval intersects the equivalent line.

### The rate of postoperative rescue analgesia

**Figure 4:**
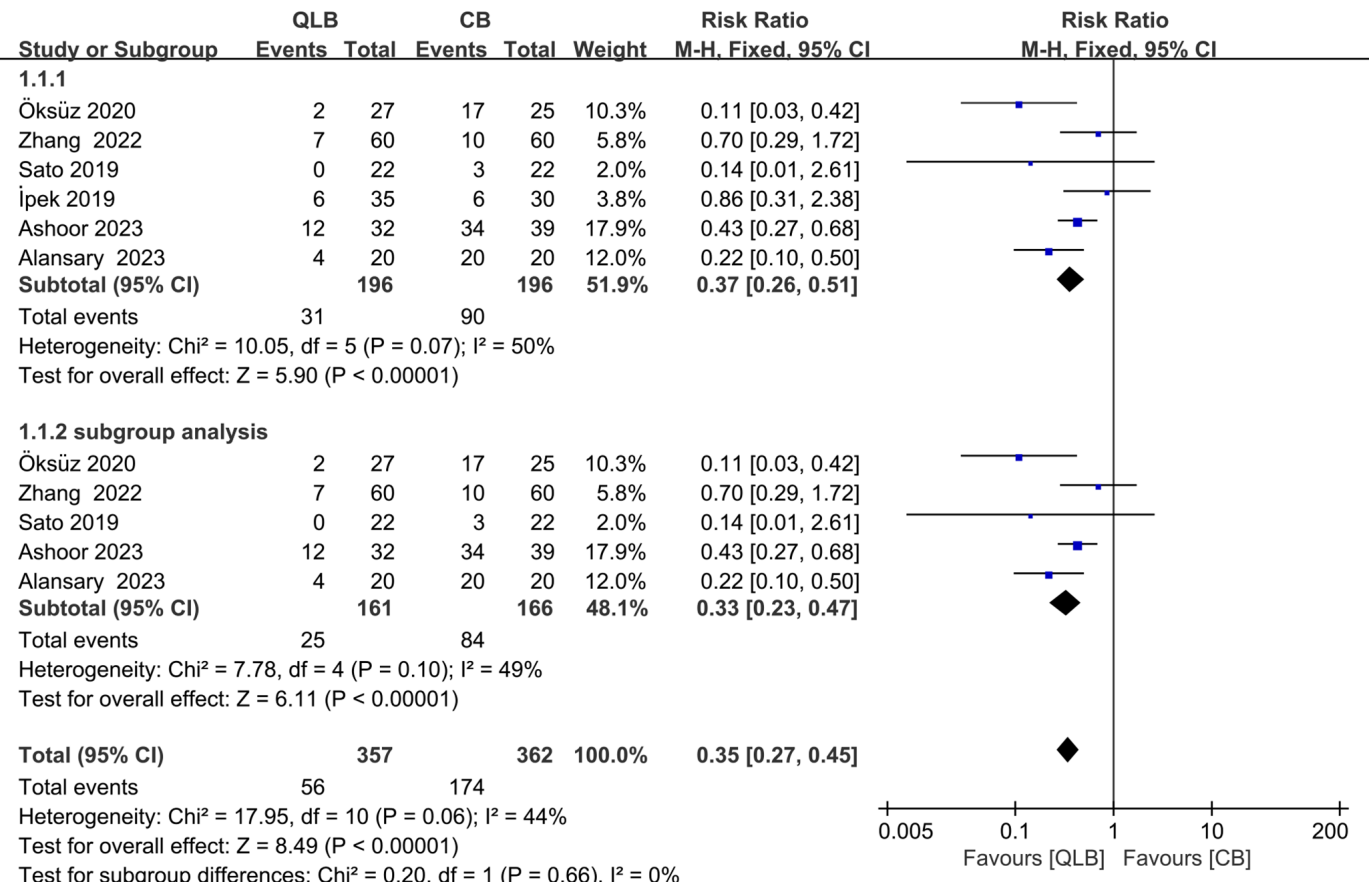
Forest plots of primary outcome of the rate of postoperative rescue analgesia.

Six trials (13, 16–19, 21) that enrolled 392 patients reported the rate of postoperative rescue analgesia. Compared with caudal analgesia, QLB showed a significant reduction in the rate of postoperative rescue analgesia. [relative risk (RR) = 0.37; 95% CI = 0.26 to 0.51; *P* < 0.01; [*I*^2^ = 50%, *P* = 0.07], Figure 4]. Among these trials, only five of them (13, 16, 17, 19, 21) utilized posterior QLB, whereas the trial by İpek et al. (18) employed lateral QLB. In order to exclude the influence of different QLB approaches on the results, subgroup analysis was conducted, and the final result was similar. [RR = 0.33; 95% CI = 0.23 to 0.47; *P* < 0.01; (*I*^2^ = 49%, *P* = 0.10), Figure 4].

### Postoperative pain scores

The postoperative pain scores at 0.5, 4, 12, and 24 h were investigated, and all studies reported pain scores at 0.5, 4, and 24 h. However, only 3 studies reported pain scores at 12 h (16, 20, 21), except for the trial conducted by Stato. M et al. (13) However, data from only four studies (13, 16, 20, 21) were analyzed in the forest plot. Because data conducted by Ashoor et al. (17) and Ipek et al. (18) were presented as graphs rather than specific values, and no original data could be obtained. In addition, the pain scores in the study conducted by Öksüz et al. (19) were reported in the form of a median, which could not be converted to the form of mean and standard deviation due to the skewed distribution of the data.

### Pain score 30 min after surgery

**Figure 5:**
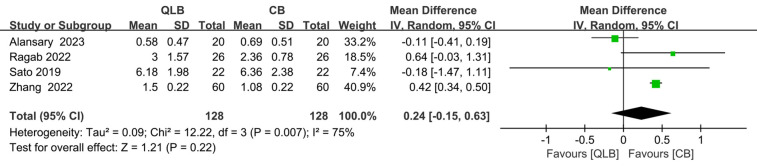
Forest plot of pain score 30 min after surgery.

At 30 min after surgery, there was no significant difference in pain scores between the two methods (SMD = 0.24; 95% CI = −0.15 to 0.63; *P* = 0.22; Figure 5), and this result was influenced by real heterogeneity (*I*^2^ = 75%, *P* = 0.007).

### Pain score 4 h after surgery

**Figure 6:**
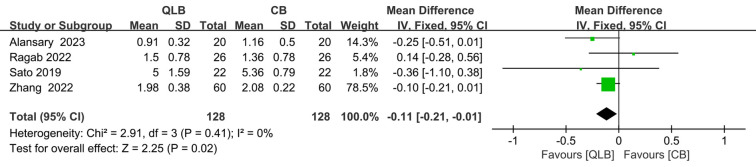
Forest plot of pain score 4 h after surgery.

At 4 h after surgery, the pain score of the QLB group was lower than that of the CB group, and the results were statistically significant [SMD = −0.11; 95% CI = −0.21 to −0.01; *P* = 0.02, (*I*^2^ = 0%, *P* = 0.41); Figure 6].

### Pain score 12 h after surgery

**Figure 7:**
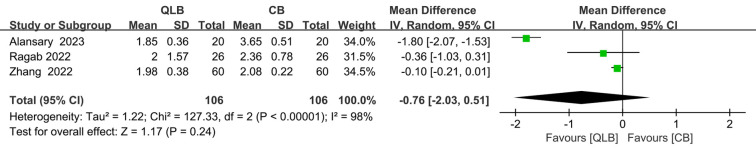
Forest plot of pain score 12 h after surgery.

At 12 h after surgery, there was no significant difference in pain scores between the two methods (SMD = −0.76; 95% CI = −2.03 to 0.51; *P* = 0.24; Figure 7), and this result was influenced by real heterogeneity (*I*^2^ = 98%, *P* < 0.01).

### Pain score 24 h after surgery

**Figure 8:**
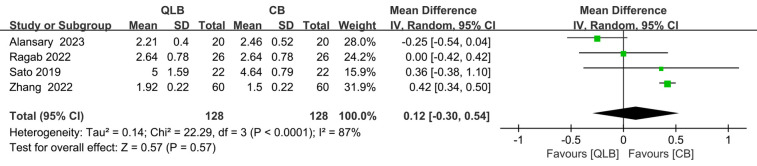
Forest plot of pain score 24 h after surgery.

At 24 h after surgery, there was no significant difference in pain scores between the two methods (SMD = 0.12; 95% CI = −0.30 to 0.54; *P* = 0.57; Figure 8), and this result was influenced by real heterogeneity (*I*^2^ = 87%, *P* < 0.01).

### Complications or side effects in the included studies

**Figure 9:**
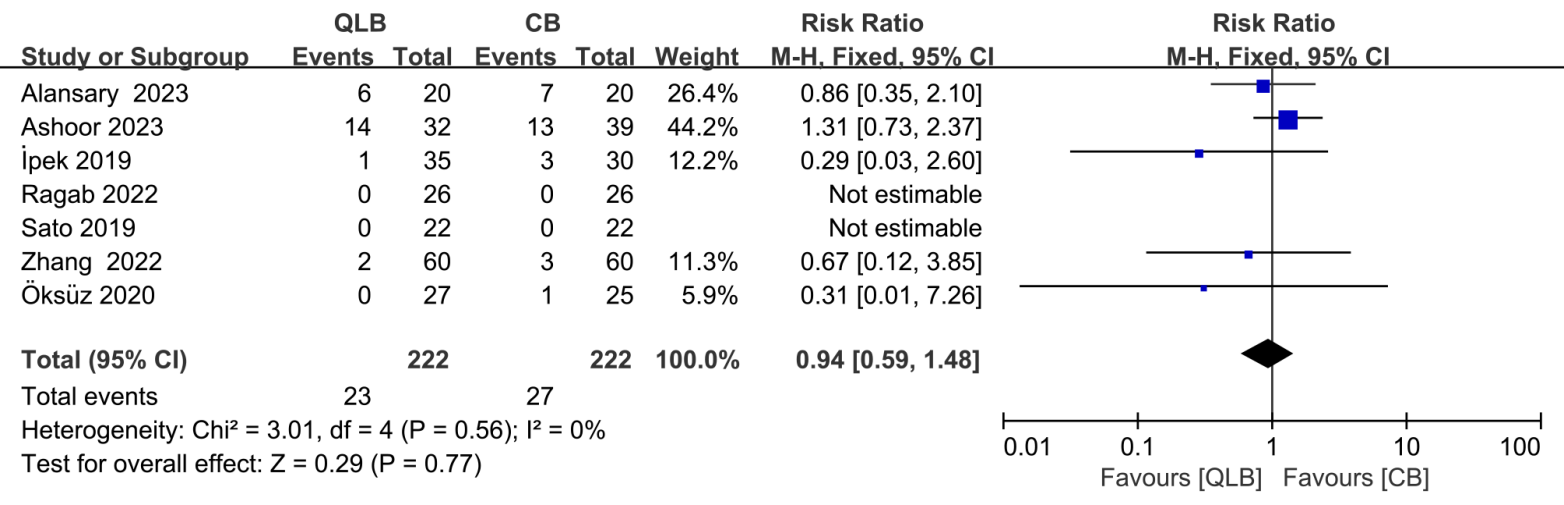
Forest plot of complications or side effects in the included studies.

All of the trials (13, 16–21) investigated the incidence of postoperative complications or by-effects, such as nausea, vomiting, hypotension, bradycardia, and urinary retention. Gözen Öksüz et al. (19) reported one case of nausea in the CB group. Celal Bulut İPEK et al. (18) identified 3 cases of postoperative urinary retention in the CB group. Tarek M. Ashoor et al. (17) detected 5 cases of urine retention, 3 cases of nausea and vomiting, 3 cases of hematoma, 2 cases of hypotension, and one case of bradycardia in the QLB group, while 9 cases of nausea and vomiting, 3 cases of urine retention, and one case of hypotension in the CB group. Yue Zhang et al. (21) reported 3 cases of nausea or vomiting in the CB group and 2 cases in the QLB group. Amin M. Alansary et al. (16) reported 7 cases of PONV in the CB group and 6 cases in the trans-incisional QLB (TiQLB) group. Makoto Sato et al. (13) and Safaa Gaber Ragab et al. (20) reported no complications. No significant difference was found between QLB and CB in the complications or side effects (RR = 0.94; 95% CI = 0.59 to 1.48; *P* = 0.77). In addition, there was no significant heterogeneity among the trials (*I*^2^ = 0%, *P* = 0.56; Figure 9).

## Discussion

To our knowledge, this was the first systematic review and meta-analysis to evaluate the postoperative analgesic effects of QLB and CB in pediatric abdominal surgeries. These results showed that compared with CB, QLB reduced the postoperative rescue analgesia rate by 0.37 during postoperative 24 h. Additionally, pain scores in the QLB group were lower than those in the CB group at 0.5 h and 24 h after surgery, and the results were statistically significant. However, it does not meet the criteria of a minimally clinically important difference, which is generally thought to be at least 1 point on the 11-point NRS (22). There were no significant differences in postoperative complications or side effects between the two groups.

Our meta-analysis further supports a previous meta-analysis (23) including CB which suggests QLB is an effective postoperative analgesia technique for pediatric patients undergoing lower abdominal surgery. In a network meta-analysis (24), the author compared the RCTs included in the study in pairs and ultimately concluded that for pediatric inguinal surgery, the first rescue analgesia time for quadratus lumborum and transversus abdominis plane blocks is the longest, and the need for rescue analgesia is the least, compared with CB, ultrasound-guided II-IHB, and other block methods. In another meta-analysis (25) shows that, when used as analgesia for hypospadias repair, CB exhibited higher pain scores 24 h postoperatively, significantly shorter analgesia duration, and greater analgesia consumption compared with peripheral nerve blocks. The author found limited data suggesting that peripheral nerve block provides better analgesic quality than CB. These conclusions align with our findings.

### Clinical implications

CB is the most frequently utilized regional anesthesia technique among children, particularly suitable for surgical interventions situated beneath the umbilicus (T10 dermatome) (26, 27). This method offers safe access to the epidural space in pediatric patients (28, 29). However, a single CB may have a relatively short duration of analgesia, only around 4 to 6 h, which can often be considered insufficient (30, 31), while placing catheters in the tail area increases the risk of infection and prevents early activity (32). The reason for its short duration is the rich distribution of blood vessels, leading to the rapid absorption of the local anesthetics (33). Furthermore, it comes with potential drawbacks such as the risk of accidental dural puncture, bladder dysfunction, and so on. Due to the above disadvantages, the application of CB is limited.

QLB is not only applicable to pediatric abdominal surgeries but it has also been proven effective in lumbar disc herniation surgeries (34), total hip replacement surgeries (35), as well as adult abdominal surgeries such as laparoscopic cholecystectomy (36) and cesarean section (37). A cadaver study (38) involving the performance of anterior QLB, using a dye, has demonstrated that QLB can induce analgesia from T10 to L4, as evidenced by the colored lumbar nerve roots and occasionally some nerves in the TAP. Additionally, other studies have indicated that anterior QLB may extend cephalad beyond previous levels, reaching T7–T12 spinal nerve roots, which could be the reason for the effectiveness of QLB (7). In adults, the mean effective duration of sensory block was 14.1 h after unilateral anterior QLB at the L4 level with 20 ml of 0.375% ropivacaine (39). Previous research (40) reported that QLB had safety features with minimal complications and/or side effects and demonstrated that QLB was the most effective technique for providing postoperative analgesia in pediatric patients undergoing lower abdominal surgery. Compared with the conventional CB or opioid analgesics in pediatric patients, QLB had a better analgesic effect and minimal side effects (41), which is consistent with our results. Compared with CB, QLB reduced the rate of rescue analgesia.

In terms of adverse reactions, although we concluded that there was no statistical difference between the QLB group and the CB group, we should pay more attention to block-related complications rather than general postoperative complications, but unfortunately, the RCTS we included did not indicate it.

### Implications for further research

The aggregated data clearly shows that QLB leads to a significantly lower rate of postoperative rescue analgesia compared to CB. As such, it is imperative to delve into the clinical significance of this difference and explore its effects on perioperative care and the overall quality of recovery. The observed trends, such as improved pain scores and a decrease in the number of patients needing additional pain relief, point to crucial research areas. This knowledge has the potential to significantly influence the development, application, and success of enhanced recovery after surgery protocols (42–44).

### Strengths and limitations

Our summary analysis is rooted in a well-considered and systematic search process, which involves independent verification and data extraction by two authors. This enables the analysis of seven studies, involving a total of 444 patients. The results derived from this large cohort are significant, as it is challenging to conduct large-scale pediatric trials required to address issues related to recovery characteristics.

However, the included studies and sample sizes were small. Small study effect bias and unpublished bias may exist. Through our results, although the difference is firm between the two groups, more studies are still needed in this area. The results of this study exhibited varying degrees of heterogeneity, prompting us to conduct a sensitivity analysis using the “one-by-one exclusion” method. Our meta-analysis revealed that a significant portion of this heterogeneity could be attributed to the article by Amin M. Alansary et al. (16). This could potentially stem from the unique approach employed in this RCT, where the author administered ultrasound-guided TiQLB to pediatric patients undergoing open renal surgery. The timing of the block, the type of surgery performed, and the specific technique used for the block all differed from those in other studies, contributing to the observed heterogeneity. In addition, in our article, the pain scores at most time points failed to reach statistical significance. This observation can likely be attributed to the comprehensive pain management protocols employed in these trials, which effectively mitigated pain levels among participants.

## Conclusion

The present meta-analysis mainly compared the analgesic effects of QLB and traditional CB, as well as side effects, to provide suggestions for selecting appropriate analgesic methods. The meta-analysis demonstrated that QLB was an effective postoperative analgesic method for the child undergoing lower abdominal surgeries and QLB might be used as an alternative to CB for pediatrics. Compared with CB, QLB provides lower pain scores in some periods or reduces the rate of postoperative rescue analgesia compared to CB. However, we identified relatively few RCTs and observed significant heterogeneity, future studies are required to provide more reliable evidence and confirm these results.

## Data Availability

The original contributions presented in the study are included in the article/Supplementary Material, further inquiries can be directed to the corresponding author.
